# Editorial: Nanomaterial design and engineering for enhanced physical therapy modalities

**DOI:** 10.3389/fbioe.2026.1878187

**Published:** 2026-06-04

**Authors:** Codina Movileanu

**Affiliations:** Ilie Murgulescu Institute of Physical Chemistry, Romanian Academy, Bucharest, Romania

**Keywords:** application of nanomaterials, biocompatibility, nanomaterial, nanoscale, physical therapy (modalities)

Physical therapy is currently at a crossroads, attempting to combine traditional methods with new ones, including those using nanomaterials. Traditional therapy modalities (stretching, manual manipulation, ultrasound, and electrical stimulation) have been used successfully over time, helping to restore mobility and relieve pain for millions of patients. Despite this, as populations age and chronic conditions such as osteoarthritis and stroke increase, these methods often fall short in terms of precision, personalization, and speed of recovery. Nanomaterials, which are tiny structures, typically less than 100 nm, are potentially revolutionary materials in physical therapy.

By designing and engineering these materials at the atomic scale, smarter, safer and more effective therapies can be unlocked, which is an urgent need (Cardoza et al., 2024; Niazi, 2026).

The effect of nanomaterials arises from their nanoscale dimensions. Such structures have applicability in various therapeutic protocols. Gold nanoparticles exhibit plasmonic effects that allow the generation of targeted heat under the effect of near-infrared light, ideal for deep tissue therapy. Carbon-based structures, such as graphene and carbon nanotubes, offer unparalleled mechanical strength and conductivity, similar to the body’s own tissues, while nanoparticle-loaded scaffolds accelerate tendon repair. Hydrogels infused with silica nanoparticles offer tunable elasticity, adapting to the patient’s movement in real time (Cardoza et al., 2024; Asadi et al., 2020; Zhu et al., 2022; Chen et al., 2024).

Wearable nanomaterials devices are considered the vanguard of this revolution. Flexible sensors embedded with zinc oxide nanowires detect muscle strain with micron-level accuracy (sensitivity up to 4% per Pa), feeding data to artificial intelligence algorithms that adjust therapy protocols on the fly. Prototypes have shown promise in stroke recovery, with haptic wearables improving motor function faster than traditional methods. Engineering these for biocompatibility–coating with polyethylene glycol to evade immune detection–ensures long-term wearability, addressing a key barrier in adoption (Cardoza et al., 2024; Nikolaeva et al., 2025; Seim et al., 2023).

Drug delivery represents another frontier. Nanomaterial carriers, such as liposomes or mesoporous silica nanoparticles, enable localized release of anti-inflammatories like ibuprofen, activated via mechanical stress from exercises. Hydrogel microsphere patches reduce OA swelling effectively through sustained release. Designing these for stimuli-responsiveness-pH-sensitive for acidic injury sites-amplifies efficacy while minimizing dosage (Chen et al., 2024; Yingyu et al., 2024; Gan et al., 2024).

Scaffolds for tissue regeneration present another challenge. In rotator cuff repair, recurrence rates range from 20% to 40%. 3D-printed nanofiber scaffolds seeded with stem cells guide precise regeneration, with clinical trials demonstrating improved healing in high-risk patients over the age of 55. Polylactic acid nanofibers reinforced with halloysite nanotubes match native tissue mechanics and degrade as tissue forms. Clinical efforts, including those at institutions exploring nanoscaffolds, are reporting strong functional outcomes (Moffat et al., 2009; Parikh et al., 2020).

The existing challenges demand rigorous engineering solutions. Biocompatibility is first: while titanium dioxide nanoparticles excel in photothermal therapy, their potential cytotoxicity requires surface functionalization. Scalability poses another hurdle; advances in microfluidics promise mass production. Regulatory hurdles loom -FDA approval lag- but streamlined pathways could help (Cardoza et al., 2024; Masarkar et al., 2024).

Ethical considerations cannot be ignored. Equity in access is paramount and open-source designs offer a blueprint. Data privacy from smart wearables demands robust encryption (Zhu et al., 2022).

Looking ahead, the synergy of nanomaterials with emerging technologies electrifies prospects. Pair nanoparticle-enhanced ultrasound with focused beam for non-invasive bone healing, as selenium nanoparticles activate pathways for defect repair. Virtual reality could overlay nanomaterial feedback, gamifying recovery (Niu et al., 2025; Chen et al., 2025).

Critics argue that long-term accumulation of nanomaterials in organs may have as yet unknown effects. The data to date contradict these views: meta-analyses show low toxicity when properly designed, with models predicting viability based on size, zeta potential, and exposure (Masarkar et al., 2024; Paula et al., 2025).

Physical therapist, materials scientists and policymakers must collaborate now.

This Research Topic has the main aim of offering possibilities to publish new research results in the basic and translational research field of the design, synthesis, and application of nanomaterials that can improve the effectiveness of physical therapy modalities. During the editorial process of this Research Topic we have appreciated that significant progress has been recently done in advanced nanomaterialbased strategies for treating inflammatory and degenerative diseases.

Hu et al. developed a macrophage-membrane-coated nanovesicle that encapsulates colchicine and exploits the body’s natural inflammatory chemotaxis to home to the infarcted heart. The system reduces multiple pro-inflammatory cytokines, limit neutrophil infiltration into the myocardium and secondary lymphoid organs and improves left ventricular function and remodeling.

Gui et al. designed a bioinspired, cardiac-targeted metal-organic framework nanozyme based on Mn-doped ZIF-8 and functionalized with atrial natriuretic peptide. The compound act as multi-enzyme mimetic that scavenges reactive oxygen species and dampens pro-inflammatory signaling, thereby improving diastolic function, reducing fibrosis/hypertrophy and ameliorating insulin-resistance-linked pathways in a murine heart-failure-withpreserved-ejection-fraction model.

Shang et al. report a synergistic photothermal-therapy platform for esophageal cancer that combines a metal-organic framework structure with photosensitizers and platinum components. Under light irradiation, the nanozymes generate heat and reactive-oxygen-species-related-activity, enabling combined photothermal and PDT-like killing of esophageal-cancer cells and enhanced tumor suppression in preclinical settings.

Zhu et al. review nanomaterial-engineered platforms (gold, carbon, liposomes, dopamine-based and others) tailored for photothermal therapy in neural tumors and neurodegenerative disorders.

All editors of this Research Topic thank all submitting authors for their work. The lead editors would like to thank all editors and reviewers for the time spent in assigning reviews, commenting on submitted manuscripts as well as reviewing. As editorial team, we hope that this special issue will prove useful in planning in nanomaterial design and engineering research in next future.

## References

[B1] Asadi S. Bianchi L. De Landro M. Korganbayev S. Schena E. Saccomandi P. (2020). Laser-induced optothermal response of gold nanoparticles: from a physical viewpoint to cancer treatment application. J. Biophot. 14 (2), e202000161. 10.1002/jbio.202000161 32761778

[B2] Cardoza J. V. Ali Z. Simon S. Thakkar D. George S. S. Isaac S. P. (2024). The role of nanoparticles in accelerating tissue recovery and inflammation control in physiotherapy practices. Cureus 16 (11), e73540. 10.7759/cureus.73540 39669817 PMC11636964

[B3] Chen J. Zhou X. Li Y. Liu T. Ding W. He F. (2024). Transforming osteoarthritis treatment: embracing hydrogel microspheres. ACS Mater. Lett. 6 (8), 3862–3882. 10.1021/acsmaterialslett.4c00668

[B4] Chen Y. Xu R. Xie B. Ma L. He Y. Liu H. (2025). Ultrasound-Driven Selenium nanoparticles realize bone defect repair through activating selenoproteins to regulate PI3K/AKT signaling pathway. ACS Nano 19 (19), 18256–18269. 10.1021/acsnano.4c18240 40338671

[B5] Gan X. Wang X. Huang Y. Li G. Kang H. (2024). Applications of hydrogels in Osteoarthritis treatment. Biomedicines 12 (4), 923. 10.3390/biomedicines12040923 38672277 PMC11048369

[B6] Masarkar A. Maparu A. K. Nukavarapu Y. S. Rai B. (2024). Predicting cytotoxicity of nanoparticles: a MetaAnalysis using machine learning. ACS Appl. Nano Mater. 7 (17), 19991–20002. 10.1021/acsanm.4c02269

[B7] Moffat K. L. Kwei A. S. Spalazzi J. P. Doty S. B. Levine W. N. Lu H. H. (2009). Novel nanofiber-based scaffold for rotator cuff repair and augmentation. Tissue Eng. Part A 15 (1), 115–126. 10.1089/ten.tea.2008.0014 18788982 PMC2809655

[B8] Niazi S. K. (2026). Nanomaterial-enabled physical rehabilitation: mechanisms, evidence, and translational pathways. Front. Rehabil. Sci. 7, 1763655. 10.3389/fresc.2026.1763655 42051601 PMC13111205

[B9] Nikolaeva A. V. Kondratev V. M. Kadinskaya S. A. Kolesina D. E. Zubov F. I. Anikina M. A. (2025). ZnO nanowire-based flexible sensors for pressure and temperature monitoring. Mater. Sci. Semicond. Process. 189, 109253. 10.1016/j.mssp.2024.109253

[B10] Niu Z. Fan Y. Yin L. Tong Y. Yao L. Ding S. (2025). Advancing nanomedicine: for bone defect repair and regeneration. Int. J. Nanomedicine 20, 15043–15062. 10.2147/IJN.S545353 41424455 PMC12717955

[B11] Parikh K. S. Josyula A. Omiadze R. Ahn J. Y. Ha Y. Ensign L. M. (2020). Nano-structured glaucoma drainage implant safely and significantly reduces intraocular pressure in rabbits *via* post-operative outflow modulation. Sci. Rep. 10, 12911. 10.1038/s41598-020-69687-4 32737340 PMC7395089

[B12] Paula A. J. Petry R. Almeida J. M. Caetano A. A. Sales J. Ferreira O. P. (2025). Large-Scale meta-analysis of nanomaterials toxicity based on natural Language processing of scientific articles. ACS Appl. Nano Mater 9 (1), 28–44. 10.1021/acsanm.5c05119 41536585 PMC12797190

[B13] Seim C. Chen B. Han C. Vacek D. Wu L. S. Lansberg M. (2023). Relief of post-stroke spasticity with acute vibrotactile stimulation: controlled crossover study of muscle and skin stimulus methods. Front. Hum. Neurosci. 17, 1206027. 10.3389/fnhum.2023.1206027 37706171 PMC10497102

[B14] Yingyu Z. Linlin Z. Liangxiao L. Yingying L. Yajun L. (2024). Research progress of nanomaterial targeted drug delivery in Osteoarthritis treatment. Chin. J. Biomed. Eng. 43 (3), 369–376. 10.3969/j.issn.0258-8021.2024.03.012

[B15] Zhu S. He Z. Ji L. Zhang W. Tong Y. Luo J. (2022). Advanced nanofiber-based scaffolds for achilles Tendon Regenerative engineering. Front. Bioeng. Biotechnol. 10, 897010. 10.3389/fbioe.2022.897010 35845401 PMC9280267

